# Fitness consequences of peak reproductive effort in a resource pulse system

**DOI:** 10.1038/s41598-017-09724-x

**Published:** 2017-08-24

**Authors:** Anni Hämäläinen, Andrew G. McAdam, Ben Dantzer, Jeffrey E. Lane, Jessica A. Haines, Murray M. Humphries, Stan Boutin

**Affiliations:** 1 Department of Biological Sciences, University of Alberta, Edmonton, AB Canada T6G 2E9; 2 Department of Integrative Biology, University of Guelph, Guelph, ON Canada N1G 2W1; 3 Department of Psychology, University of Michigan, Ann Arbor, MI 48109 USA; 4 Department of Ecology and Evolutionary Biology, University of Michigan, Ann Arbor, MI 48109 USA; 5 Department of Biology, University of Saskatchewan, Saskatoon, SK Canada; 6 Department of Natural Resource Sciences, McGill University, Ste-Anne-de-Bellevue, QC Canada

## Abstract

The age trajectory of reproductive performance of many iteroparous species features an early - life increase in performance followed by a late - life senescent decline. The largest contribution of lifetime reproductive success is therefore gained at the age at which reproductive performance peaks. Using long term data on North American red squirrels we show that the environmental conditions individuals encountered could cause variation among individuals in the “height” and timing of this peak, contributing to life history variation and fitness in this population that experiences irregular resource pulses. As expected, high peak effort was positively associated with lifetime reproductive output up to a high level of annual effort. Furthermore, individuals that matched their peak reproductive effort to an anticipated resource pulse gained substantial fitness benefits through recruiting more offspring over their lifetime. Individual variation in peak reproductive effort thus has strong potential to shape life history evolution by facilitating adaptation to fluctuating environments.

## Introduction

Reproductive performance typically changes non-linearly throughout life due to age-specific trade-offs between resource allocation into self-maintenance and reproduction^1, 2^. A pattern commonly seen in iteroparous species features increasing reproductive output due to an increasing reproductive investment^2–4^ and experience^5^ until a peak in prime age, followed by decreasing reproductive performance in late life due to senescence (refs 6–8, but see ref. 9).

In a profile of age-specific reproduction, the age at which reproductive output peaks is, by definition, the life phase that has the highest influence on lifetime reproductive output as a larger proportion of an individual’s offspring are produced at that age than at any other. An obvious peak in the age trajectory of reproductive performance could result from individuals maximizing their investment in a specific reproductive event, with lower effort expended in other events. The age at which investment in current reproduction peaks may vary due to intrinsic quality, condition or reproductive strategy^10–13^ or the environment^14^. The realized age trajectories of reproductive performance can, therefore, vary widely among individuals^15–17^ as well as species^9^.

To maximize their lifetime reproductive success, a major fitness component, individuals should attempt to maximize their payoffs for reproductive effort by adjusting their resource allocation according to the potential fitness payoffs. Flexibility in effort might consequently be expected to be most adaptive in fluctuating environments, where reproductive effort has different fitness outcomes among reproductive opportunities^18–20^ and iteroparity typically evolves^21, 22^. The highest fitness should follow from maximizing effort when the probability of success is the highest, for example when the environmental conditions forecast high juvenile survival^23–26^ or the parent’s ability to produce and rear offspring (e.g. resource availability^27–29^; competition^30^). The fitness payoffs may also depend on the individual’s internal state such as body condition^31, 32^, or an interaction of intrinsic and extrinsic conditions^33^. The optimal age profile of reproductive effort may thus differ among individuals^11^, but individual differences in the timing of maximum reproductive effort and investment in the peak phase, its fitness consequences, or the implications for life history evolution have not been previously studied (but see refs 14, 34 and 35 for studies of related life history components). Even less is known about individual differences in, and the adaptive value of, matching effort to prevailing conditions.

We propose that the variability in extrinsic breeding conditions an individual is likely to experience during its lifespan should influence the optimal adjustment of maximum reproductive effort. The alternative hypothesis would be relatively constant reproductive effort throughout life. Consequently, when fitness is likely to be improved more by an increased effort under beneficial intrinsic or extrinsic conditions than by a constant effort among reproductive events, adjustable effort should be selected for. Under less variable conditions, a more constant effort might be beneficial and thus the potential for extreme allocation is less likely to be adaptive. The actual expression of effort is likely constrained by individual quality or condition, and contrasting selective pressures that confer fitness benefits under suboptimal conditions may constrain the maximum possible effort.

We examined the fitness consequences of variable maximum reproductive effort in an iteroparous species, the North American red squirrel (*Tamiasciurus hudsonicus*). Our study population experiences strongly fluctuating resource availability, population density and juvenile recruitment success due to their reliance on seeds of a masting tree species, the white spruce (*Picea glauca*)^36^. Juvenile overwinter survival is high in mast years^37^ when more juveniles are able to cache sufficient food to survive their first winter^38^. Red squirrels increase their reproductive efforts in the months immediately preceding mast seed production^36^, implying that they can accurately predict masting via some extrinsic cue, and respond to the high anticipated fitness payoffs before gaining access to additional food. This elevated reproductive effort thus occurs *before* the spruce seed is available, representing a potentially adaptive, and likely energetically costly, behaviour.

Given the high recruitment success in mast years^36, 37^, we predicted that maximizing individual effort in a mast year should increase the mother’s lifetime reproductive success. We tested for fitness consequences of maximum reproductive effort by estimating how individual differences in maximum effort (maximum annual pup production) predict lifetime reproductive success (lifetime total number of recruited offspring). We predicted that the magnitude of peak reproductive effort should correlate positively with lifetime reproductive output, and that females should exhibit their peak effort in a mast year if they encountered one in their lifetime. If females were not adaptively adjusting their reproductive effort, reproductive peaks would be no more likely in mast years than in non-mast years.

We show that a high maximum reproductive effort, particularly when effort was timed to coincide with anticipated high fitness payoffs, generally predicts higher lifetime reproductive success up to a biologically feasible level of effort. Despite heterogeneity among individuals in the timing of the reproductive peak, the age at which peak effort was expressed did not influence lifetime reproductive success. We thus provide the first evidence that characteristics of the reproductive peak phase have fitness consequences independent of age-specific reproductive allocation, and that the adaptiveness of such adjustments may be mediated by environmental conditions.

## Methods

### Data collection

We monitored the complete life histories of individually-marked female North American red squirrels (*Tamiasciurus hudsonicus*, hereafter ‘‘red squirrels”) near Kluane National Park in the southwest Yukon, Canada (61°N, 138°W) from 1987 through 2013 via regular live-captures and tracking of reproduction. Detailed descriptions of the study system and data collection protocols are provided elsewhere^39^. We recorded lifespan to the year by regular live-capture and via behavioural observations of all territory owners throughout life, as resighting probability is close to 1^26^. Reproductive and survival data were collected for squirrels born in 1986-2009, excluding females that were still alive in 2014. We included in our analyses 548 females that gave birth to at least one pup in their lifetime. To control for the potential effect of females that only reproduced in one year (N = 114), we repeated the analyses excluding those females. The conclusions were similar with this restriction (see Supplementary Information (SI)). Thus, as excluding these females might disguise alternative strategies induced by the environment or introduce selective disappearance issues^40, 41^, we included them in the analyses.

We located litters and marked pups individually within 3 days of birth^39^. We recorded the number of pups each female produced each reproductive season (“**Annual effort**”), including pups from litters that were lost prior to weaning as well as multiple litters produced in mast years^36^. We then determined which pups survived their first winter to sexual maturity (“**Recruitment**”) by recapturing all territory owners. This is a good estimate of juvenile survival, as dispersal out of the study site is rare and a territory with a food cache is required for winter survival^38, 42, 43^. The maximum annual number of pups a female produced in her lifetime was considered her “**Peak effort**”, and the peak timing (“**Peak age**”) was considered to be the age at which they first achieved this maximum effort. The “fitness payoff” of reproductive effort was quantified as the annual number of recruited offspring, or summed over the lifetime to quantify lifetime reproductive success, a major component of individual fitness. The total number of pups produced was used as an alternative measure of reproductive success as was done previously^39, 44, 45^ because this metric is less affected by offspring “quality” independent of the mother^46^. The results for most analyses of lifetime pup production are presented in SI owing to space restrictions.

We monitored food availability annually via white spruce cone counts in late summer after cones had matured but prior to caching by red squirrels (detailed description in ref. 47). Cones were counted annually on one side of the top 3 m of approximately 250 spruce trees distributed across the study area, ln(x + 1)-transformed and averaged. The resulting cone production index well reflects food availability as spruce seed is the main food resource for squirrels in the population^48^. Four years in the study period (1993, 1998, 2005, 2010) were identified as mast years following previously established criteria^49^.

### Statistics

#### Fitness consequences of peak reproductive effort

We first tested whether lifetime reproductive success was associated with peak effort. The lifetime number of pups produced was modeled using a Generalized Linear Mixed-Effects Model (GLMM) with a Poisson error distribution and log link, and lifetime number of recruits was modeled with a GLMM with a negative binomial error distribution (due to overdispersion). **Maximum effort** (highest annual # pups produced by the female) was included in each model. We included a quadratic as well as a linear term of maximum effort to test whether the effect of maximum effort on lifetime reproductive output reaches a plateau at some optimum level of effort. Such an optimum might be predicted if the maximum number of annual pups is limited by the accumulating costs of offspring production, or by the number of offspring a female could feasibly rear in a breeding season. A linear and quadratic term of peak age was included to assess the significance of peak timing on lifetime reproductive output. As age at first reproduction and lifespan are known to influence female fitness in this species^39^, we included “**Primiparity**” (binary variable indicating whether a female produced their first litter as a yearling or at a later age) and **Lifespan** in the model. The mother’s year of birth (“**Cohort**”: N = 24 overall, but N = 23 for females that experienced a mast year, as no reproducing females born in 1987 experienced a mast) was added as a random effect to control for cohort effects^26^. Removing the single maximum effort of 15 pups from the analyses did not change the outcome, and the case was retained in the analyses.

In terms of lifetime pup production, there is an implicit prediction of a positive relationship with peak effort, as pups produced over the lifetime can never be fewer than the maximum annual number of pups produced. A slope of 1 would be expected if females bred only once, their only attempt thus being equal to their peak effort as well as their lifetime pup count. We tested whether the observed relationship between peak effort and lifetime pup production exceeded this null expectation through a procedure of resampling only from the range where lifetime pups ≥ maximum annual pups^50^ (see SI for details), and ran the best model for lifetime pup production (Poisson GLMM) for 1000 iterations of simulated data.

#### Effect of a resource pulse on the optimal timing of peak effort

To test whether females were more likely to maximize their reproductive effort when the anticipated reproductive payoffs were highest, we restricted the data to the 260 reproducing females that experienced a mast year in their lifetime. For investigating individual likelihood of responding to a mast year with maximum effort, we analysed these females’ annual reproductive records (N = 1020 records/260 individuals) to test whether individuals were more likely to exhibit peak effort in a mast year than in other years of their life. This was done by assigning each year of a female’s life a status of 1 if she achieved her maximum reproductive effort in that year, and 0 for all non-peak years. Similarly, each year was assigned as either mast year or non-mast year. We first modeled the binary response variable “**Peak year**” (whether or not the annual record was the year in which the individual exhibited their peak effort) using a binomial GLMM with a logit link. We then tested whether females produced more offspring in mast years than in non-mast years, using a Poisson GLMM to model annual pups, and a negative binomial GLMM to model annual recruits. Each of these models included the predictor variable “**Mast year**” (whether or not the annual record was from a mast year), and squirrel identity nested within cohort as a random effect.

We then tested whether matching effort to environmental cues influenced fitness. We quantified the fitness effect of maximizing effort in a mast year (binary variable “**Mast peak**”: whether peak year was the same year as the mast year) on the lifetime number of recruits with a negative binomial GLMM and log-link. Primiparity and lifespan were included as covariates and cohort as a random effect.

We addressed potential limitations for maximizing effort in a mast year with a binomial GLMM. We predicted that very young and old females would be physiologically less capable of responding to the mast year with maximum capacity, and thus tested for the linear and quadratic effect of “**Age when encountering mast**” on whether or not the individual’s peak effort occurred in a mast year (Mast peak as response variable). High reproductive effort early in life might hamper maximizing effort in a mast year, thus we also included the “**Pre-peak rate of reproduction**” (number of pups produced/year before the peak age) as a fixed effect. Cohort was included as a random effect.

To determine the costs of peak “height” (magnitude of peak effort), we examined the effect of peak effort on post-peak survival and reproduction, depending on whether the peak occurred in a mast year. We analyzed the effects of peak reproduction on “**Post-peak lifespan”** (years of life after peak age) with a Linear Mixed-Effects Model, and **“Post-peak reproduction”** (total number of pups produced after peak age) with a Poisson GLMM and log-link. Both models included the terms mast peak, peak effort, and their interaction, as well as peak age. Cohort was included as a random effect.

We fitted all models with the R package *lme4* ^51^ and used Satterthwaite’s approximation in *lmerTest* ^52^ to calculate P-values. We reduced all models using backward selection to exclude non-significant variables (P < 0.05), starting with interaction and polynomial terms. We scaled all continuous predictor variables (within each analysis) to a mean of zero and unit variance to facilitate interpretation of relative effect sizes. We present both the scaled and back-transformed estimates in the main text. We computed the 95% confidence intervals (CI) associated with model parameters using bootstrapping implemented with *confint*.*merMod* in package *lme4*. We report results based on the final models, and provide all full model outputs in SI. All analyses were conducted with program R version 3.3.1^53^. See SI for further details on the modelling approach and model diagnostics.

### Data accessibility statement

The datasets supporting this article are available at https://figshare.com/articles/Hamalainen_SciRep_fitness_consequences_of_peak_effort_data_xlsx/5307382/1.

### Ethics statement

All animal studies were conducted in accordance with the Canadian Council on Animal Care Guidelines and Policies with approval from the Animal Care and Use Committee for Biosciences for the University of Alberta.

## Results

### Fitness consequences of maximum reproductive effort

**Figure 1:**
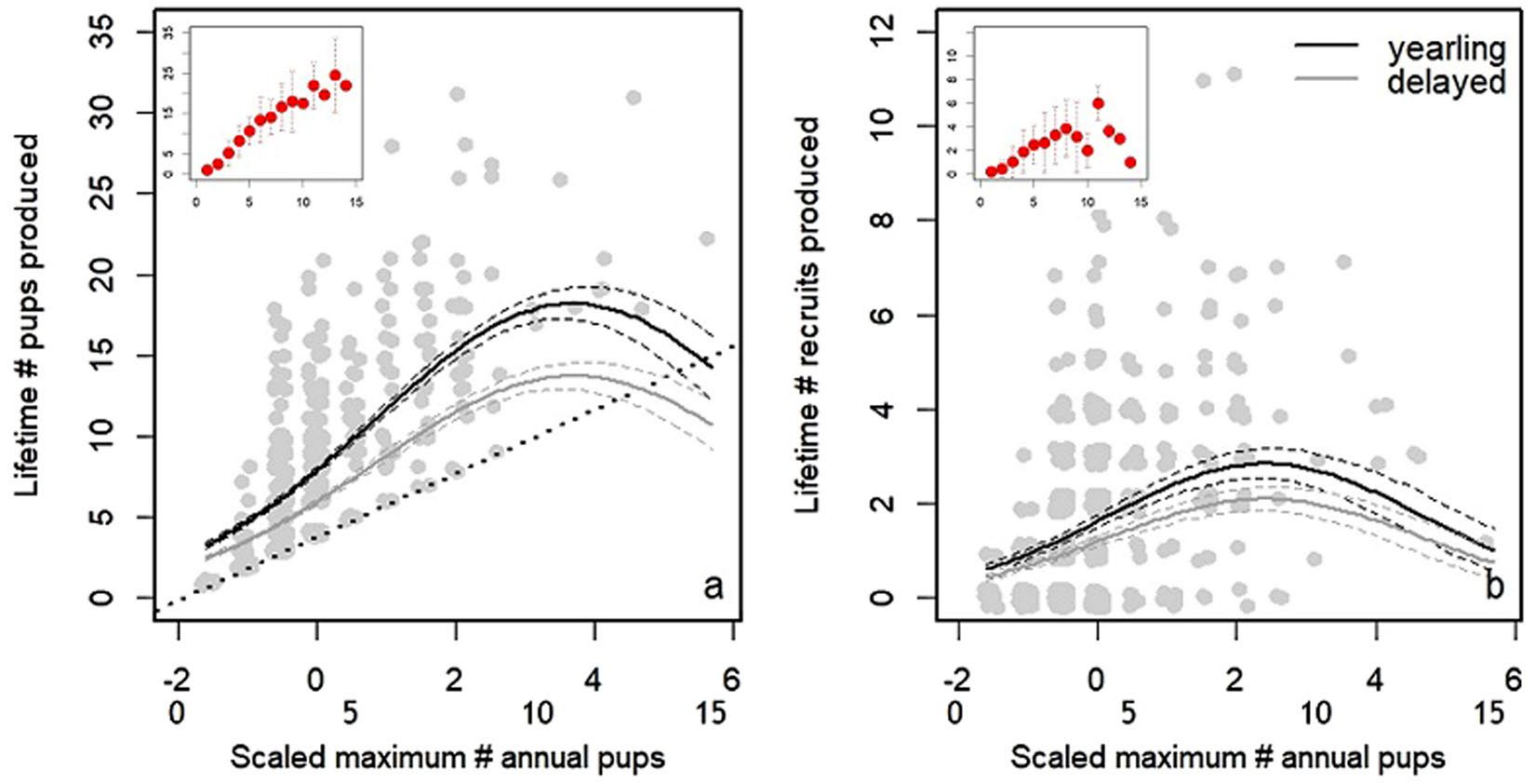
Maximum annual # of pups (maximum effort) has a significant effect on lifetime number of (**a**) pups and (**b**) recruits produced by red squirrel females. Lifetime reproductive success is improved by an increasing maximum effort up to (**a**) 11 pups for lifetime number of pups, and up to (**b**) 9 pups for lifetime number of recruits. A plateau or even declining reproductive success follows if maximum effort exceeds this threshold. Note that the models are based on scaled maximum # annual pups (x-axis top row), with the corresponding scale of actual maximum number of pups (1–15) indicated below, on the bottom row of the axis. Gray points indicate raw data with a small amount of jitter introduced to show overlapping points. The lines show the predicted effect of maximum effort on lifetime reproductive output (correcting for lifespan and age at primiparity), from the model for females that began breeding as yearlings (solid black line) or at age 2 years or above (solid gray line). Standard errors represented by the surrounding dashed lines. The dotted line in (**a**) indicates the null hypothesis of slope 1, as lifetime # pups must be equal to or larger than the maximum annual # pups produced. The insets summarize the raw data, with the points indicating means and the associated error bars the standard deviation of lifetime reproductive output for each level of peak effort.

As predicted, a high peak effort predicted higher lifetime reproductive output, with an increase in lifetime number of pups and recruits with increasing peak effort up to a high threshold level (Fig. 1). The lifetime number of pups born to a female increased with their maximum effort up to 11 pups (peak effort with largest effect on lifetime pup production from the model predictions = 11.2 pups, maximum = 15 pups). A maximum effort of 12 or more pups did not further increase lifetime success, as suggested by the negative quadratic trend in the effect of maximum effort on lifetime pup production, although it should be noted that a maximum effort of >11 pups was only achieved by 5 females (Table 1, Fig. 1a). Note that the predicted (but not observed) lifetime reproductive success at very high levels of effort drop below the plausible range (Fig. 1a), suggesting a strong effect also of lifespan and/or age at primiparity on lifetime pup production in females with very high maximum effort.

**Table 1:** The effect of maximum reproductive effort (maximum annual # pups) on lifetime reproductive success in terms of total number of pups produced over the lifetime (Poisson GLMM), and total number of recruited offspring over the lifetime (Negative binomial GLMM), based on the best models.

		β	Back-transformed estimate	CI 2.5%	CI 97.5%	Z	P
Pups*	Intercept	1.780		1.719	1.832	39.7	<0.001
	**Max. annual pups**	**0.454**	**4.885**	**4.784**	**4.980**	**18.2**	**<0.001**
	**Max. annual pups** ^2^	**−0.061**	**−4.123**	**−4.154**	**−4.096**	**−8.2**	**<0.001**
	Peak age	0.022	2.223	−2.228	2.278	0.8	0.408
	**Peak age** ^2^	**−0.037**	**−2.241**	**−2.267**	**−2.218**	**−3.3**	**<0.001**
	**Lifespan**	**0.419**	**4.031**	**3.965**	**4.083**	**22.5**	**<0.001**
	**Primiparity** ^**‡**^	**0.284**		**0.214**	**0.355**	**7.7**	**<0.001**
Recruits^†^	Intercept	0.184		−0.014	0.342	2.09	<0.001
	**Max. annual pups**	**0.462**	**4.901**	**4.699**	**5.122**	**7.27**	**<0.001**
	**Max. annual pups** ^2^	**−0.096**	**−4.191**	**−4.276**	**−4.119**	**−4.47**	**<0.001**
	**Lifespan**	**0.542**	**4.245**	**4.092**	**4.381**	**12.6**	**<0.001**
	**Primiparity** ^**‡**^	**0.306**		**0.135**	**0.471**	**3.62**	**<0.001**

Significant results in bold. Back-transformed estimates and associated confidence intervals (CI) as well as the scaled estimate are given for parameters that were scaled for the analysis, and untransformed intervals are provided for un-scaled terms. *Poisson GLMM, N = 548 females; Random effect: Cohort (N = 24) variance = 0.000 ± 0.016. ^†^Negative binomial GLMM, N = 548 females; Random effect: Cohort (N = 24) variance = 0.070 ± 0.265; Negative binomial dispersion parameter = 11.2. ^‡^Reference value: delayed; i.e. positive value indicates a higher lifetime reproductive success of females that began breeding as yearlings.

The results of the resampling analysis indicate that despite the obligate relationship where minimum lifetime # pups = maximum annual effort, the effect of maximum effort on lifetime reproductive effort was higher than expected by chance, as the estimate from the real model (original data: β linear term of scaled peak effort = 0.454 (CI: 0.402–0.503)) exceeded all estimates from resampling the plausible range (lifetime pups ≥ peak effort; simulated data: mean β = 0.019, range β = −0.028–0.072. See SI for further details.).

A higher number of pups produced in the peak year also predicted higher lifetime recruitment success up to a threshold of a peak effort of 9 pups (highest predicted lifetime number of recruits was gained with 8.7 pups based on model predictions; Table 1, Fig. 1b), with a slight decline in success associated with very high peak effort. This suggests that very high annual maximum effort may be associated with relatively high offspring mortality, leading to lower recruitment success, but it should be noted that this result is driven by only 10 females that exhibited a maximum effort of >9 pups.

The strong positive effect of peak effort on lifetime recruitment success suggests that timing peak effort to a year with high recruitment success (mast year) may drive this relationship. However, repeating the analysis with only those females that never experienced a mast in their lifetime (N = 288 females from 18 cohorts) indicated a similar relationship for the effect of maximum effort on lifetime recruitment success (Linear effect of maximum pups: β = 0.34 (CI: 0.113–0.568), z = 2.73, P = 0.006; SI: Table S2). Thus, while peak effort in a mast year conferred maximal fitness benefits, the effect of high peak effort on lifetime reproductive output was not limited to the resource pulse effect.

An earlier age at primiparity and a longer lifespan also increased lifetime reproductive output in terms of both pups and recruits (Table 1). The age at peak reproductive output ranged from 1–7 years (median = 2, mean = 2.2 years). The best model for lifetime pup production indicated that a peak occurring at a very late age had a negative effect on lifetime pup production (Table 1), but a visual examination of the data suggests this effect to be very small (SI: Figure S1). The age at peak was not associated with lifetime recruitment success (linear and quadratic terms both non-significant at P > 0.3, SI: Table S1, Figure S1). Taken together, the magnitude of peak effort appears to have stronger fitness consequences than peak age.

### Effect of a resource pulse on the optimal timing of maximum effort

Females that experienced a mast year produced on average nearly twice as many pups in mast years than in non-mast years (non-mast = 2.08 ± 1.86 pups, mast = 3.66 ± 2.66, β = 0.569 (CI: 0.494–0.654), N = 1020 annual breeding records/260 individuals), and their recruitment success was three times higher in mast years (non-mast = 0.38 ± 0.72 recruits; mast = 1.38 ± 1.40 recruits, β = 1.315 (CI 95%: 1.120–1.502); mean proportion of pups recruited: non-mast = 20%, mast = 41%). See SI for model details.

Out of the 260 breeding females that encountered a mast year in their lifetime, 54% (140) exhibited peak reproductive effort in the mast year. Given that these females had up to 7 (median: 3) reproductive events in their lifetime and only 4 mast years occurred in 1986–2013, the mast events were highly influential for individual life histories. Indeed, at individual level, maximum effort was significantly more likely to occur in a mast year than in any other year of a female’s lifetime (effect of mast on likelihood of peak occurring in that year: β = 1.932 (CI: 1.622 – 2.280), z = 11.920, P < 0.001).

The likelihood of peak reproduction coinciding with a mast year (“Mast peak”) increased when the resource pulse occurred earlier in life (linear effect of age at mast year: β = −1.228 (CI: −2.303–0.703), z = −3.41, P = 0.001, quadratic term of age n.s., see SI), suggesting that older individuals had a reduced ability to respond to a mast year. Reproductive effort before the peak year did not influence the likelihood of maximizing effort in a mast year (pre-peak rate of reproduction: β = 0.091 (CI: −0.182–0.347), z = 0.759, P = 0.448).

Females that exhibited their maximum effort in a mast year had a higher lifetime reproductive success, as they recruited on average 30% more offspring than females that encountered a mast but peaked in a non-mast year (mast peak: 2.97 ± 2.20; non-mast peak: 2.08 ± 1.95 recruits; effect of mast peak on recruitment success controlling for lifespan, primiparity and cohort: β = 0.492 (CI: 0.304–0.663), z = 5.40, P < 0.001). Due to the higher recruitment rate in mast years, they also had a higher proportion of pups produced over their lifetime survive to recruitment (mast peak: 30.7 ± 22.2%, non-mast peak: 18.2 ± 15.6%).

For those individuals that experienced a mast year, survival in years after the peak year was lower for those females that peaked in a mast year (average post-peak lifespan: 1.39 ± 1.30 years) than those that peaked in a non-mast year (1.91 ± 1.58 years), but the number of offspring produced after the peak did not significantly differ between females that peaked in a mast year or a non-mast year (Table 2). Thus, the fitness benefits of exhibiting peak reproduction in a mast year were associated with a survival cost but no reproductive cost.

**Table 2:** Costs of peak reproductive effort.

		β	Back-transformed estimate	CI 2.5%	CI 97.5%	Statistic	P
Post-peak survival*	Intercept	2.2245		1.727	2.721	9.12	<0.001
	**Mast peak**	**−0.3715**		**−0.656**	**−0.062**	**−2.35**	**0.019**
	**Peak age**	**−0.6551**	**−2.969**	**−3.175**	**−2.753**	**−7.65**	**<0.001**
Post-peak reproduction^†^	Intercept	1.0494		0.732	1.321	6.95	<0.001
	**Max pups**	**0.314**	**4.614**	**4.491**	**4.727**	**8.06**	**<0.001**
	**Peak age**	**−0.4317**	**−2.706**	**−2.819**	**−2.617**	**−9.57**	**<0.001**

Predictions for post-peak effort (total # of pups produced after the peak) and survival (post-peak lifespan) depending on the level of maximum effort, age at peak and whether or not maximum effort was expended in a mast year. Post-peak survival was analysed with a Gaussian LMM (test statistic = t), post-peak reproduction with a Poisson GLMM (test statistic = z). Back-transformed estimates and associated confidence intervals (CI) as well as the scaled estimate are given for parameters that were scaled for the analysis, and untransformed intervals are provided for un-scaled terms. N = 260 individuals from 23 cohorts. *Random effect, cohort: variance = 0.925 ± 0.962. ^†^Random effect, cohort: variance = 0.422 ± 0.65.

## Discussion

Our study demonstrates that variation in the magnitude and timing of peak reproduction, an often-overlooked life history phase in iteroparous species, can have substantial fitness consequences. We show that the maximum reproductive effort, i.e. the “height” of the peak of the age trajectory of reproductive effort, is positively associated with lifetime reproductive success up to a high level of effort. When females exhibited their reproductive peak effort in a year when juvenile survival was very high due to a resource pulse (mast years), they reaped substantial fitness gains for this effort despite their reduced survival. Despite the high fitness benefits of maximizing effort in a mast year, not all females that experienced a mast year achieved their maximum effort in that year, at least partially due to age-related constraints in maximizing reproductive effort. Individual ability to optimize effort among reproductive opportunities under fluctuating conditions is therefore an important driver of life history variation.

### Reproductive Peak as a Life History Trait

While our results corroborate previous evidence in red squirrels for the fitness benefits of a long lifespan^39^ and an early age of first reproduction^39, 54^, peak reproductive performance further explained variation in lifetime reproductive success. Considering the height and timing of the peak of the age trajectory in combination with the reproductive lifespan is therefore a potentially powerful way of profiling complete individual life histories particularly in species with large variation in potential reproductive output across breeding events. An increasing peak effort improved lifetime reproductive output up to a very high annual effort. The plateau or even a decline in the relationship between maximum effort and lifetime reproductive output at very high levels of maximum effort suggests that there is a feasibility limit to annual pup production, where fitness benefits continue to be gained. As the sample size is very limited for females with exceptionally high maximum effort, this result should be interpreted with caution, but it indicates that females that produce a very high number of pups in their peak year experience substantial pup mortality because a relatively small proportion of their offspring recruit. Very high annual numbers of pups are only likely to occur in mast years, where females frequently re-cycle after losing a litter, and the breeding season is extended. Some females are subsequently able to successfully wean two litters in mast years^36^.

Population-level estimates of peak reproductive age^6–8^ can differ from individual-level patterns as population trends tend to be biased by the selective mortality of lower “quality” individuals^40, 41, 55^, and through adjustments in individual reproductive effort to environmental conditions. While most individuals in this study achieved their peak reproductive output before 3 years of age (mean: 2.2 years), the population level reproductive performance of red squirrels peaks between ages 3–5 years depending on the trait^26, 39, 56^. While it has been recognized that heterogeneity in life histories must be accounted for to model population-level patterns and evolutionary processes^24, 57, 58^, variability in complete life history profiles among individuals have rarely been examined empirically prior to this study (but see refs 34, 35 and 59). Given the high variability in the timing and magnitude of individual peak reproduction, and the associated fitness effects, we recommend assessing peak reproductive effort at individual-level. This approach could lead to a better understanding of the microevolutionary processes operating through flexible reproductive adjustments, and facilitate more accurate modeling of population-level processes. Promising analytical methods to further dissect such variation^60, 61^ could permit a closer examination of these processes in wild populations.

It is noteworthy that the age at which peak effort occurred did not influence fitness in terms of lifetime recruitment success, whereas the height of the peak had a substantial fitness effect. This implies that flexibility in response to intrinsic or extrinsic cues or constraints was more important in shaping individual reproductive profiles than age itself. This concurs with the proposition that variable age trajectories may be produced by state-dependent investment^11^ (see also ref. 62). The timing and height of the peak relative to other reproductive events likely varies by species. For example, semelparous species have a single reproductive event, possibly because maximum reproductive effort is under such strong selection that further reproductive events cease to be attempted^63^. To our knowledge the only other study that has examined both peak height and timing in an iteroparous species found that neriid fly (*Telostylinus angusticollis*) males that attained a high condition due to a higher nutrient availability early in life, peaked earlier but did not attain a higher peak than low-condition males^34^. The earlier peak of high-condition males was associated with accelerated senescence and a shorter lifespan, but the fitness effects of these strategies remain to be tested.

#### Adaptive adjustment of reproductive effort to environmental conditions

The relative importance of peak reproduction increased in mast years, suggesting that the ability to mobilize resources for reproductive allocation under high-payoff conditions has substantial fitness consequences. Despite the relative rarity of mast years compared to non-mast years individual females were much more likely to exhibit peak reproductive effort in a mast year than any other year. This provides additional evidence of the remarkable flexibility in the squirrels’ response to predicted juvenile survival^30, 36, 48, 64^. A number of other species also appear to make reproductive decisions based on environmental cues (e.g. refs 18 and 65–67), while others do not seem to adjust their reproductive effort to environmental conditions^68–70^.

While a high reproductive peak had no apparent reproductive costs, we found evidence of a trade-off between survival and maximum effort, as higher peak effort was associated with reduced survival. The trend for a very high maximum effort to have a reduced impact on lifetime reproductive success (suggested by the negative quadratic trend of maximum effort for lifetime pup and recruit production) might also indicate costs of high reproductive effort, but due to the limited number of females exhibiting very high maximum effort, this possibility remains speculative. Reproductive allocation tends to incur higher costs under demanding environmental conditions^71, 72^, and current reproduction should be prioritized when future reproductive or survival prospects are low relative to the value of the current reproductive event^3, 12, 73^. Red squirrels invest high effort in mast years with limited resources, as they breed before the maturing mast cone crop is available to them. Despite being presumably highly energetically demanding, a mast year represents exceptionally beneficial conditions for squirrels through the high likelihood of offspring recruitment. Timing peak reproductive allocation in a mast year should be adaptive despite the energetic and survival costs, as experiencing another event with such high payoffs is extremely unlikely (0.01%, i.e. 6 females in our data set experienced 2 mast events as adults).

Evidence of trade-offs between early-life fecundity and survival or reproduction later in life are frequently documented^17, 54, 74–77^; (but see ref. 78). Our results suggest that variation in age-specific reproductive effort and thus, relationships between life history stages, may sometimes be created by short-term responses to environmental conditions that are at least partially independent of age. Such responses are adaptive if environmental cues can be used to predict immediate reproductive success (e.g. via relative survival prospects of adults vs. juveniles^19^). In red squirrels, flexibility likely has greater fitness benefits than a fixed strategy because of the unpredictable nature of masts at the inter-annual scale. This pattern is not uniform across systems, however, as reproductive effort appears to be relatively evenly distributed among reproductive opportunities in some birds despite varying fitness payoffs or survival prospects^68, 69^. These differing patterns may result from different optimal life histories due to characteristics of the environment, or stem from a differing availability of reliable cues of anticipated breeding success. In the absence of reliable cues, adjustments to environmental cues may be constrained to minimize risks of erroneous adjustment. A conservative strategy is likely favored by long-lived species when the level of fluctuation is relatively small, as suggested by the risk-averse adjustments of reindeer (*Rangifer tarandus*) to changing food availability^79^.

#### Individual variability in ability to adjust reproductive effort

Heterogeneity in breeding propensity and reproductive allocation may arise via varying individual quality or physiological state in interaction with environmental conditions (evidence obtained mainly from long-lived birds^69, 80, 81^). Variability among individuals in adjusting to prevailing conditions remains poorly understood, but evidence suggests that the ability to adjust effort optimally may depend on individual quality^33, 82^, energetic status^32^ or exploration tendency^83^. While, at the individual level, females in this study were much more likely to maximize their reproduction in a mast year if they experienced one, only 54% of females that experienced a mast year achieved their reproductive peak in a mast year. As the likelihood of such adaptive adjustment decreased with age, senescence (e.g. declines in reproductive physiology or resource acquisition ability) appeared to impair individual ability to adaptively maximize reproductive effort (see also ref. 84). Further investigation into the constraints to maximizing effort are warranted, and could provide clues to interactions of the environmental cues with individual condition, phenotypic quality, or alternative strategies such as offspring quality-quantity trade-offs or different parenting styles.

Environmental cues of low predicted payoffs for breeding may invoke prolonged self-maintenance via reduced allocation in current reproduction^39, 85^, (see also ref. 86). An alternative strategy of a low pace of reproduction over multiple years may therefore be a useful strategy for making the best of a bad situation for those red squirrels that did not experience a mast year, or experienced one late in life. This is likely reflected in the fitness benefits of an earlier onset of reproduction and a longer lifespan (ref. 39, this study). Fitness is often determined by an interaction of phenotypic quality or age with the experienced environment^33, 87^, and a longer lifespan might increase the likelihood of experiencing an exceptionally good year. Theoretical work supports this strategy, suggesting that iteroparity and delayed development should both be under positive selection in stochastic environments^22^.

#### Future directions

Matching peak reproductive effort with beneficial environmental conditions may be under especially strong selection in resource pulse systems, but the mechanisms likely operate to some extent in all iteroparous species in which the predicted success varies among reproductive opportunities. Interesting opportunities for testing the significance of varying reproductive allocation relative to individual qualities and environmental variation may be afforded e.g. by the comparative study of species or populations that show tactics of annual reproductive allocation ranging from semelparity to iteroparity, such as certain salmonids^88, 89^. Systems with such a range of tactics would be especially useful for dissecting the effects of the environment on life history strategies through a simultaneous assessment of the determinants of the timing of first reproduction, peak reproductive effort, and terminal investment.

Juvenile survival is a major component in determining red squirrel population growth^39^, thus traits improving recruitment success (via maternal care, environmental conditions or their interaction) are likely under strong selection in this species. Maximum annual number of offspring therefore serves well to identify the reproductive peak in species with substantial variation in potential annual offspring number. Other traits may be more relevant in species that are longer-lived, or produce a single offspring per cycle. An assessment of effort relative to other individuals in the population, effort in terms of energy expenditure and allocation, or the proportion of lifetime success attributable to the peak event will be useful in understanding the broader significance of the reproductive peak across species. A recent analytical approach proposed by Authier *et al*.^62^ would be an interesting way to assess the relevance of individual quality in determining responsiveness to the environment. Such methods could be used to further explore to what extent neutral vs. directional selective processes^62, 90^ generate the variation among individuals in the timing and intensity of maximum effort.

## Conclusions

We show for the first time that maximum annual reproductive effort significantly influences individual fitness in an iteroparous species. We therefore advocate the inclusion of the reproductive peak as a component of the life history phenotype in studies of life history evolution. Individual variation in the maximum reproductive effort and flexibility in effort have potential to shape life history evolution by facilitating adaptation to fluctuating environments.

## Electronic supplementary material

Supplementary information
