# Supplementary material for: Fitness consequences of peak reproductive effort in a resource pulse system
Source: Sci Rep. 2017 Aug 24;7:9335. doi: 10.1038/s41598-017-09724-x (PMC5571191; doi:10.1038/s41598-017-09724-x)
Supplement: Supplementary file 1 — Supplementary information [file 41598_2017_9724_MOESM1_ESM.pdf]

Supplementary Information for

Fitness consequences of peak reproductive effort in a resource pulse system

Anni Hämäläinen, Andrew G. McAdam, Ben Dantzer, Jeffrey E. Lane, Jessica A. Haines, Murray M. Humphries, Stan Boutin

Supplementary information contents

Modelling approach and model diagnostics 2
- Model diagnostics 2
- Data completeness 2
- Restricting the data to females that reproduced more than once 3

Supplementary results 3
- Fitness consequences of peak reproductive effort 3
  - Recruits full model 4
  - Effect of peak on fitness in females that never experienced a mast year 4
  - Effect of maximum effort on recruitment success, excluding females that only reproduced once 5
  - Effect of peak age on lifetime reproductive output 6
  - Resampling analyses 7
- Effect of timing peak effort to a mast year 8
  - Likelihood of exhibiting maximum reproductive effort depending on mast occurrence 8
  - Effect of mast year on annual offspring production 8
  - Effect of maximizing effort in a mast year on lifetime number of offspring 9
  - Limitations of maximizing effort in a mast year 10
  - Costs of peak reproductive effort 10

References 12

Modelling approach and model diagnostics

Model diagnostics

A third of all females that attempted reproduction in the time frame of the study (31%, 247/795 individuals) produced zero pups over their lifetime (maximum number of pups produced by a single female: 31). If those individuals with zero success were excluded from the modeling, the modal number of pups produced over the lifetime for successful females was 3 pups (114 individuals). In terms of recruitment success, 209 (38%) of the 548 females that produced at least one pup in their lifetime had zero pups survive to recruitment age. The range for lifetime recruits produced by a female was 0-11 recruits, the mode being 1 recruit for successful females.

As a result of these distributions, a Poisson model could not be employed for lifetime recruit data. Overdispersion was confirmed by calculating the sum of squared Pearson residuals, over the residual degrees of freedom: SSQ/rdf=1.10, and a negative binomial model thus provided a better fit to the data (likelihood ratio test comparing Poisson & Negative binomial models of recruit production: resulted in Deviance=6.41, P=0.011, indicating the negative binomial model as superior). For lifetime pup production, no overdispersion was detected (SSQ/rdf=0.54), and a Poisson model provided a sufficient fit to the data.

Variance Inflation Factors (VIF) were calculated (excluding random effects) to ensure that multicollinearity was not an issue, and all VIF values were below sqrt(vif)<2. The computation of dfbeta-values confirmed that influential cases were unlikely to bias our estimates (dfbetas all ≤ 1), and all cases were retained in the analyses.

The information theoretic approach to model selection (results not shown) using AICc weights supported the outcome of the backward selection procedure and identified the same variables as influential for lifetime reproductive output.

Data completeness

The data were restricted to females born between 1986 and 2009 and last encountered a year before the end of the study period (i.e. died in 2013 or earlier), to ensure we only included complete lifetime records for adult females. We only included females of known birth year that survived to recruitment (age 1 year) and whose breeding attempts were subsequently successfully monitored. Our results did not change when restricting the data to females born at least 8 years (maximum lifespan) prior to the end of the study period (i.e. before 2006, excluding 89 females), implying that bias towards shorter lifespans in the latter years did not skew our results.

Restricting the data to females that reproduced more than once

The average reproductive events/female in females that experienced a mast year=3.1, median=2, range=1-8, in females that did not experience a mast year=1.7, median=1, range 1-6.

Females that only produced pups in one year (N=114) might produce bias in the data because their only successful reproductive event (attempted reproduction in other years, i.e. pregnancies that were not carried to term do not contribute to the pup count) makes up their entire lifetime reproductive output and is typically associated with a short lifespan. However, even if a female only breeds once, her effort at that time is expected to depend on the prevailing environmental conditions and is therefore informative for the lifetime adjustment of effort. Excluding these females might disguise alternative strategies induced by the environment (though our results do not directly indicate the presence of such strategies) and introduce selective disappearance issues (van de Pol & Verhulst 2006; Nussey *et al.* 2011). To assess the influence these females had on the data, we repeated the lifetime reproduction models using only females that reproduced in more than one year. The results were qualitatively very similar to those from the full data set and the conclusions remained the same (Tables S3 and S4).

Supplementary results

Fitness consequences of peak reproductive effort

The full model for recruitment success (Table S1) suggested that the age at peak was not associated with lifetime recruitment success, hence the quadratic and linear terms of Peak age were removed from the model one after the other. The quadratic term was initially included to assess the possibility that achieving peak effort in prime adulthood would be most adaptive, following previous population-level accounts of highest reproductive success at age 3-5 (Descamps *et al.* 2007, 2008; McAdam *et al.* 2007).

For lifetime pup production, all terms of the full model were retained in the final model as the quadratic term of Peak age was indicated as statistically significant (main text, Table 1).

Recruits full model

Table S1: The effect of peak reproductive effort (maximum annual # pups) on lifetime reproductive success (lifetime # recruited offspring) based on the full model (Negative binomial GLMM).

	**β**	**CI 2.5%**	**CI 97.5 %**	**z**	**P**
Intercept	0.205	0.004	0.364	2.31	0.021
**Max. annual pups**	**0.498**	**0.372**	**0.636**	**7.30**	**<0.001**
**Max. annual pups^2**	**-0.103**	**-0.151**	**-0.065**	**-4.66**	**<0.001**
**Primiparity (ref. delayed)**	**0.253**	**0.063**	**0.437**	**2.71**	**0.007**
**Lifespan**	**0.566**	**0.467**	**0.650**	**12.29**	**<0.001**
Peak age	-0.086	-0.200	0.046	-1.32	0.186
Peak age^2	0.012	-0.045	0.059	0.46	0.642
GLMM, N=548 females; Random effect: Cohort (N=24) variance=0.063±0.251; Negative binomial dispersion parameter: 11.08					

Effect of peak on fitness in females that never experienced a mast year

Repeating the analysis of recruitment success as a function of maximum effort (full model) with only those females that never experienced a mast in their lifetime suggested a similar effect of peak effort on lifetime recruitment success (Table S2), although the model failed to converge.

Table S2: The effect of peak reproductive effort (maximum annual # pups) on lifetime reproductive success (lifetime # recruited offspring) for females that did not experience a mast year in their lifetime, based on the full model (Negative binomial GLMM).

	**β**	**CI 2.5%**	**CI 97.5%**	**z**	**P**
Intercept	-0.330	-0.6	-0.027	-2.17	0.030
**Max. annual pups**	**0.340**	**0.132**	**0.612**	**2.73**	**0.006**
**Max. annual pups^2**	**-0.075**	**-0.161**	**-0.023**	**-2.08**	**0.037**
Primiparity (ref. delayed)	-0.086	-0.473	0.262	-0.46	0.643
**Lifespan**	**0.788**	**0.621**	**0.936**	**8.55**	**<0.001**
Peak age	-0.161	-0.43	0.087	-1.17	0.243

Peak age^2	0.001	-0.088	0.099	0.02	0.983
GLMM, N=288 females; Random effect: Cohort (N=18) variance=0.115±0.338; Negative binomial dispersion parameter: 17421					

Effect of maximum effort on recruitment success, excluding females that only reproduced once

In terms of both pups and recruits produced over the lifetime, the results were overall qualitatively similar when excluding data for females that only reproduced once in their lifetime. Two exceptions were found, however, as the significant trend for the quadratic effect of peak age on lifetime pup production was no longer significant (but trend in the same direction approached significance; Table S3) as for the full data set. In contrast, the linear term of peak age on recruit production was not significant for the full data set, but became so in the restricted data (trends in the same direction in full and restricted data; Table S4).

Pups

Table S3: The effect of maximum reproductive effort (maximum annual # pups) on lifetime reproductive success (lifetime # recruited offspring) for females that reproduced in more than one year, based on the full model (Negative binomial GLMM).

	**β**	**CI 2.5%**	**CI 97.5%**	**z**	**P**
Intercept	1.979	1.920	2.036	64.8	<0.001
**Max. annual pups**	**0.434**	**0.386**	**0.484**	**16.7**	**<0.001**
**Max. annual pups^2**	**-0.061**	**-0.077**	**-0.044**	**-7.1**	**<0.001**
**Primiparity (ref. delayed)**	**0.296**	**0.219**	**0.371**	**7.9**	**<0.001**
**Lifespan**	**0.337**	**0.301**	**0.369**	**18.9**	**<0.001**
Peak age	-0.014	-0.059	0.030	-0.6	0.554
Peak age^2	-0.021	-0.043	-0.001	-1.9	0.057
Poisson GLMM, N=434 females; Random effect: Cohort (N=24) variance=0.002±0.042					

Recruits

Table S4: The effect of maximum reproductive effort (maximum annual # pups) on lifetime reproductive success (lifetime # recruited offspring) for females that reproduced in more than one year, based on the full model (Negative binomial GLMM).

	**β**	**CI 2.5%**	**CI 97.5%**	**z**	**P**
Intercept	0.453	0.274	0.614	5.63	<0.001
**Max. annual pups**	**0.467**	**0.338**	**0.604**	**7.08**	**<0.001**
**Max. annual pups^2**	**-0.108**	**-0.157**	**-0.063**	**-4.53**	**<0.001**
**Primiparity (ref. delayed)**	**0.273**	**0.084**	**0.442**	**3.05**	**0.002**
**Lifespan**	**0.420**	**0.338**	**0.493**	**10.3**	**<0.001**
**Peak age**	**-0.122**	**-0.228**	**-0.006**	**-2.29**	**0.022**
Peak age^2	0.040	-0.013	0.083	1.64	0.102

Negative binomial GLMM, N=434 females; Random effect: Cohort (N=24) variance=0.046±0.214; Negative binomial dispersion parameter: 17.71

Effect of peak age on lifetime reproductive output

A high peak effort (maximum annual # pups) predicted higher lifetime reproductive output in terms of pups as well as recruits (see main text Table 1), but the age at which peak occurred had a much weaker effect on lifetime reproductive success. Peak effort exhibited late in life was associated with a lower lifetime number of pups produced over the lifetime (non-significant linear, but significant negative quadratic effect of age at peak) (main text Table 1), however based on visual inspection of the data this effect appears to be quite weak (Figure S1A). No effect of peak age was found on recruitment success (Table S1, Figure S1B).

Many individuals produced a most of their pups in a single reproductive season (78% (425/548) of all females had a peak year where >50% of lifetime pups were produced in a single year), and 21% (114/548) of females only experienced one successful reproductive event.

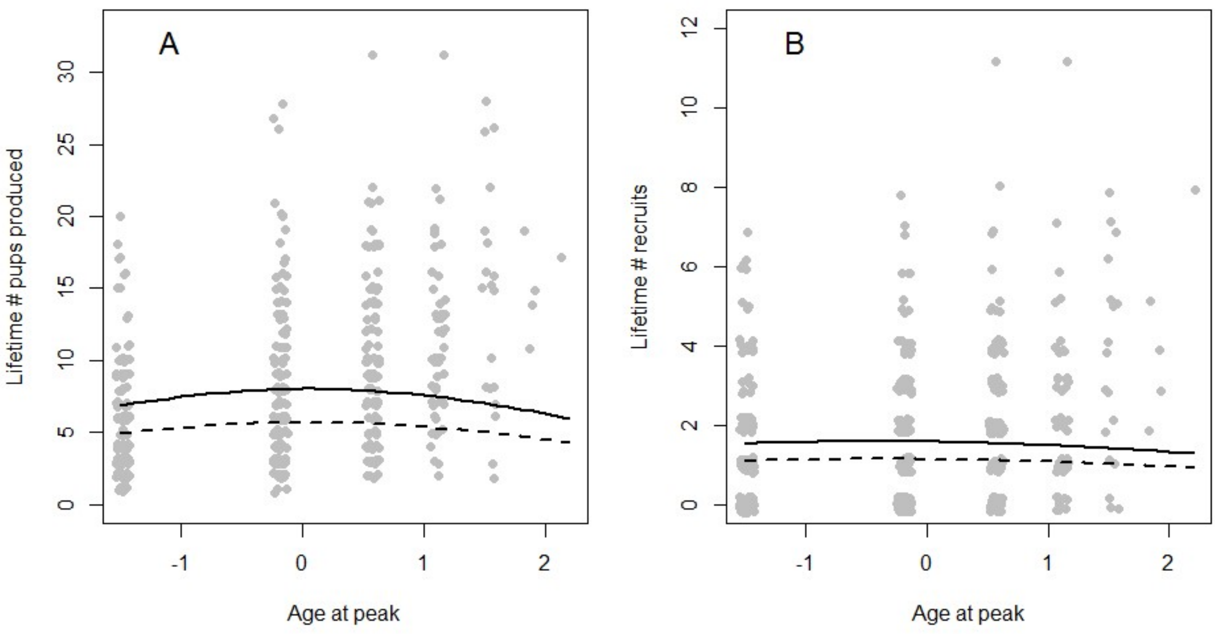

Figure S1. A) Age at peak (centred and scaled) was weakly associated with lifetime # pups produced (very slight decline in predicted lifetime pups if peak occurs late in life, quadratic term of peak age significant). B) Age at peak was not associated with lifetime recruitment success. Grey points indicate raw data, with a small amount of jitter added to show overlapping points. Solid lines indicate prediction for females that began breeding as yearlings, dashed line females that delayed their first reproduction.

Resampling analyses

Lifetime pup production could theoretically reach a similar level following a strategy of a single year of high effort, or cumulatively via multiple years of breeding at a lower intensity. As lifetime pup production is at minimum equal to the individual's maximum annual effort, a positive relationship is the null prediction: a slope of 1 would be expected if females bred only once, their only attempt thus being equal to their maximum annual effort as well as their lifetime reproductive output. Therefore, we assessed the effect of this null prediction on our model outcome for lifetime pup production (Poisson GLMM; main text Table 1) by resampling lifetime pup production with replacement from the possible range of values (where lifetime pups ≥ maximum annual pups (peak effort); *sensu* (Lane *et al.* 2011)). Resampling of the data with this restriction evaluates the proportion of the reproductive output additional to the minimum peak reproduction required to produce the observed lifetime pup output. We ran the models for 1000 iterations of simulated data in order to produce confidence intervals and compare the results from our real data with the simulated data.

There is a clear relationship between the maximum number of annual pups and the total number of pups produced in the lifetime (main text Table 1, Figure 1), i.e. maximum effort is closely linked to lifetime reproductive output. The significance of this relationship was confirmed by the resampling analysis. All predicted estimates based on the simulated data from resampling only the possible range of values for lifetime pups (≥ maximum annual # pups, scaled values) were lower (mean β= 0.019, range β=-0.028 - 0.072) than the observed values based on the real data (effect of peak effort on lifetime # pups based on original data: β= 0.454 (CI:0.402-0.503)).

In terms of recruits, the relationship is less rigid: although the number of recruits over the lifetime could potentially only be as high as the total number of pups produced in the lifetime (regardless of the contribution of the maximum annual effort to lifetime # pups), the recruitment success was typically much lower than maximum effort (Figure 1), and only 8 individuals recruited all of the pups they produced (up to 4 young). We thus tested the null hypothesis that maximum effort does not influence recruitment success by resampling lifetime reproductive success (total recruits produced) using the full range of lifetime recruits (i.e. not restricting the minimum recruitment success = maximum effort) and ran the best model (negative binomial GLMM; Table 1) for each of the 1000 iterations of simulated data.

The simulated data consistently predicted a much lower effect size (mean = -0.002; range= -0.060 - 0.048; scaled values) than was observed in our data (Observed estimate for effect of peak effort from real data β= 0.462). This refutes the null hypothesis, confirming that a high annual maximum effort strongly predicts a higher number of recruited offspring.

Effect of timing peak effort to a mast year

Likelihood of exhibiting maximum reproductive effort depending on mast occurrence

Maximum effort was significantly more likely to occur in a mast year than in any other year for those females that encountered a mast year in their lifetime. A binomial GLMM of 1020 annual records/260 females/23 cohorts revealed a significant positive effect of mast occurrence on a female exhibiting peak effort in a given year: β =1.932 (CI:1.622 – 2.280), z=11.92, P<0.001. Intercept: -1.834 (CI: -2.065- -1.661), z= -17.37, P<0.001, random effects: squirrel id/cohort <0.001, cohort <0.001.

Effect of mast year on annual offspring production

Pups

Females produced more pups in mast years (β=0.569 (CI: 0.492 – 0.651), z=13.64, P<0.001) than in the non-mast years in which they reproduced (Poisson GLMM, N=1020 annual records/ 260 breeding females that experienced a mast year, intercept: β =0.692 (CI: 0.564 – 0.817), z=-10.90, P<0.001, random effects: squirrel id/cohort: variance=0.068±0.261, cohort: 0.060±0.245.

Recruits

Females produced more recruits in mast years (β =1.315 (CI:1.105-1.494), z=13.42, P<0.001) than in the non-mast years in which they reproduced. Negative binomial GLMM, N=1020 annual records/ 260 breeding females that experienced a mast year, intercept: β =-1.044 (CI:-1.291 - -0.857), z=-10.05, P<0.001, random effects: squirrel id/cohort: variance<0.001, cohort: 0.110±0.331; Negative binomial dispersion parameter: 1.772.

Effect of maximizing effort in a mast year on lifetime number of offspring

Pups

Females that exhibited their maximum effort in a mast year also produced more pups over their lifetime (Table S5).

Table S5: The effect of achieving peak reproductive effort in a mast year vs. in a non-mast year (Mast peak) on lifetime # pups produced, based on the full model (Poisson GLMM) for females that encountered a mast year in their lifetime.

	**β**	**CI 2.5%**	**CI 97.5%**	**z**	**P**
Intercept	1.663	1.520	1.800	22.18	<0.001
**Mast peak (ref. peak in non-mast year)**	**0.161**	**0.077**	**0.244**	**3.77**	**<0.001**
**Primiparity (ref. delayed)**	**0.327**	**0.230**	**0.423**	**6.84**	**<0.001**
**Lifespan**	**0.438**	**0.391**	**0.495**	**16.61**	**<0.001**
Poisson GLMM, N=260 females;<br>Random effect: Cohort (N=23) variance=0.061±0.247					

Recruits

Females that exhibited their maximum effort in a mast year had a higher lifetime recruitment success (Table S6).

Table S6: The effect of achieving peak reproductive effort in a mast year vs. in a non-mast year (Mast peak) on lifetime # recruits produced, based on the full model (Negative binomial GLMM) for females that encountered a mast year in their lifetime.

	β	CI 2.5%	CI 97.5%	z	P
Intercept	0.007	-0.268	0.255	0.05	0.96
**Mast peak (ref. peak in non-mast year)**	**0.492**	**0.326**	**0.682**	**5.40**	**<0.001**
**Primiparity (ref. delayed)**	**0.441**	**0.229**	**0.629**	**4.48**	**<0.001**
**Lifespan**	**0.403**	**0.306**	**0.504**	**7.66**	**<0.001**
Negative binomial GLMM, N=260 females;<br>Random effect: Cohort (N=23) variance=0.074±0.272<br>Negative binomial dispersion parameter: 23.03					

Limitations of maximizing effort in a mast year

The likelihood of peak reproduction coinciding with a mast year increased when the resource pulse occurred earlier in life, but reproductive effort before the peak year did not influence the likelihood of maximizing effort in a mast year (Table S7).

Table S7: The effects of age when encountering a mast and breeding rate prior to the peak year on the likelihood of achieving maximum reproductive effort in a mast year, based on the full model (Binomial GLMM) for females that encountered a mast year in their lifetime.

	β	CI 2.5%	CI 97.5%	z	P
Intercept	-0.295	-0.860	0.218	-1.16	0.245
**Age in mast year**	**-1.228**	**-2.303**	**-0.703**	**-3.41**	**<0.001**
Age in mast year^2	-0.445	-1.165	-0.054	-1.66	0.096
Rate of reproduction before peak year	0.091	-0.182	0.347	0.74	0.458
Binomial GLMM, N=260 females;<br>Random effect: Cohort (N=23) variance=0.043±0.207					

Costs of peak reproductive effort

Table S8: Costs of peak reproductive effort. Predictions from full models for post-peak effort (total # of pups produced after the peak) and survival (post-peak lifespan) depending on the level of maximum effort, age at peak and whether or not maximum effort was expended in a mast year. Post-peak survival was analysed with a Gaussian LMM (test statistic =t), post-peak reproduction with a Poisson GLMM (test statistic =z).

		β	CI 2.5%	CI 97.5%	Statistic	P
Post-peak survival [a]	Intercept	2.121	1.593	2.654	8.34	<0.001
	**Mast peak**	**-0.443**	**-0.753**	**-0.137**	**-2.73**	**0.007**
	Max pups	0.177	-0.009	0.381	1.80	0.073
	**Peak age**	**-0.705**	**0.882**	**-0.543**	**-7.91**	<0.001
Post-peak reproduction [b]	Intercept	1.090	0.809	1.402	7.32	<0.001
	Mast peak	-0.104	-0.261	0.045	-1.29	0.20
	**Max pups**	**0.324**	**0.253**	**0.400**	**8.22**	<0.001
	**Peak age**	**-0.425**	**-0.525**	**-0.450**	**-9.43**	<0.001

N=260 individuals from 23 cohorts
a) Random effect, cohort: variance=0.966±0.983
b) Random effect, cohort: variance=0.387±0.622

References

1.Descamps, S., Boutin, S., Berteaux, D. & Gaillard, J.-M. (2007). Female red squirrels fit Williams' hypothesis of increasing reproductive effort with increasing age. *J. Anim. Ecol.*, 76, 1192–1201

2.Descamps, S., Boutin, S., Berteaux, D., McAdam, A.G. & Jean-Michel, G. (2008). Cohort effects in red squirrels: the influence of density, food abundance and temperature on future survival and reproductive success. *J. Anim. Ecol.*, 77, 305–314

3.Lane, J.E., Kruuk, L.E.B., Charmantier, A., Murie, J.O., Coltman, D.W., Buoro, M., *et al.* (2011). A quantitative genetic analysis of hibernation emergence date in a wild population of Columbian ground squirrels. *J. Evol. Biol.*, 24, 1949–1959

4.McAdam, A.G., Boutin, S., Sykes, A.K. & Humphries, M.M. (2007). Life histories of female red squirrels and their contributions to population growth and lifetime fitness. *Ecoscience*, 14, 362

5.Nussey, D.H., Coulson, T., Delorme, D., Clutton-Brock, T.H., Pemberton, J.M., Festa-Bianchet, M., *et al.* (2011). Patterns of body mass senescence and selective disappearance differ among three species of free-living ungulates. *Ecology*, 92, 1936–1947

6.van de Pol, M. & Verhulst, S. (2006). Age-Dependent Traits: A New Statistical Model to Separate Within- and Between-Individual Effects. *Am. Nat.*, 167, 766–773
